# Collaborative research and the co-production of knowledge for practice: an illustrative case study

**DOI:** 10.1186/s13012-016-0383-9

**Published:** 2016-02-20

**Authors:** Janet Heaton, Jo Day, Nicky Britten

**Affiliations:** Institute of Health Research, University of Exeter Medical School, St Luke’s Campus, Exeter, Devon EX1 2SL UK

**Keywords:** Collaborative health research, Co-production, Knowledge translation

## Abstract

**Background:**

In 2008, the National Institute for Health Research (NIHR) began funding a major 5-year pilot research programme of translational research in England, establishing nine ‘Collaborations for Leadership in Applied Health Research and Care’ (CLAHRCs). A number of evaluations were carried out to examine whether or not the various collaborations worked as intended and why. In this paper, we examine what the theory of co-production adds to understanding of processes of knowledge creation and translation we observed in one of the CLAHRCs.

**Methods:**

A case study of a successful knowledge translation project was identified from our wider realist evaluation of the mechanisms of closer collaboration at play in the CLAHRC. In the project, a computer simulation model of an emergency pathway for acute ischaemic stroke was built to explore if and how the time between the onset and treatment of the condition could be minimised by redesigning the pathway. The aim of the case study was to improve our understanding of the nature and workings of the mechanisms of closer collaboration that were associated with the more successful projects by examining the relevance of the theory of co-production. Qualitative methods of analysis were used to explore the fit between the mechanisms of closer collaboration we observed in the realist evaluation and the principles of co-production we identified from the literature.

**Results:**

We found a close fit between the nine mechanisms of closer collaboration at work in the project and the principles of co-production (active agents; equality of partners; reciprocity and mutuality; transformative; and facilitated). The successful style of collaborative working exemplified by the project was consistent with a strong form of co-production.

**Conclusions:**

In our view, the theory of co-production provides useful insights into what it is about the qualities of collaborative working that inspire the requisite mechanisms for generating knowledge that is translated into practice. The theory provides a potentially useful basis for future knowledge translation programmes and projects in applied health research in a range of contexts.

## Background

The ways in which applied health research is undertaken in England has changed in recent years [1]. Previously, researchers based in universities carried out studies with little involvement of those who commissioned, provided or used health services. This system generated knowledge that was not always relevant to or used by the latter groups [2, 3]. Increasingly, the government and other funders of health care research have sought to close the gap in the production and utilisation of knowledge, encouraging innovation and promoting evidence-based policy and practice in the National Health Service (NHS).

To this end, the National Institute for Health Research (NIHR) began funding a major 5-year pilot research programme in 2008. The programme enabled universities and NHS Trusts to form local research partnerships, called ‘Collaborations for Leadership in Applied Health Research and Care’ (CLAHRCs). Initially, nine CLAHRCs were funded across England at a cost of £90 million to NIHR, matched by their partner organisations [1]. Their objectives were to conduct high-quality research, implement the findings and increase capacity for applied health research (AHR) in their geographical areas. Each CLAHRC addressed a distinct set of research themes and priorities that reflected the needs of their local population and the partners’ interests and expertise (e.g. [4]). At the end of the pilot, 13 new or geographically reconfigured CLAHRCs were funded by NIHR for another 5 years, to work alongside Academic Health Science Networks (AHSNs) that had been established in a related bid to accelerate innovation and mobilise knowledge in the NHS [5].

The various approaches adopted by the nine original CLAHRCs were examined in a series of external evaluations funded by the NIHR Service Development and Organisation (SDO) programme [6–9]. Several CLAHRCs also included formative internal evaluations embedded within them [10]. This paper stems from our internal evaluation of the NIHR CLAHRC for the South-West Peninsula (PenCLAHRC). In its pilot form, PenCLAHRC was a partnership between two universities and 13 NHS Trusts in the far south-west of England (subsequently expanded to cover a wider area and more NHS trusts in 2014–2018). Its leaders sought to establish a system for the design, conduct and implementation of AHR on a collaborative basis. The system was built on the notion of ‘Engagement by Design’^©^ whereby researchers worked closely with clinicians and managers in the NHS, as well as patients and the public, at all stages in the research process [11]. It was believed that this closer collaboration would lead to more successful knowledge translation.

### The original evaluation

In our evaluation, we adopted a realist approach [12] to examine members’ theories about closer collaboration and whether or not this approach worked as intended and why. We were particularly interested in identifying the mechanisms by which PenCLAHRC’s emphasis on closer collaboration influenced participants’ reasoning and behaviour in the contexts of the different projects it supported. Based on an examination of four PenCLAHRC projects that had made variable progress towards their goals, we identified nine mechanisms of closer collaboration that made a difference to the projects’ success. These mechanisms are summarised in Table 1 and have been described in depth elsewhere in a report of the overall findings of the evaluation [13].

**Table 1:** Nine mechanisms of closer collaboration

Mechanisms of closer collaboration	
M1: Local end-user driven—Local end-users are placed at the heart of AHR. They are involved in driving research, so that it focuses on real-life issues that are relevant and important to them, and throughout the research life cycle	
M2: Meeting of minds—End-users and researchers find a common and coherent objective around which they coalesce. Their commitment and enthusiasm is matched with strategic support from their respective organisations	
M3: Knowledge appetite—End-users and researchers are open and receptive to melding different forms of knowledge and expertise. This includes clinicians’ knowledge of routine clinical practice, patients’ experiential knowledge, and researchers’ methodological expertise. Each recognises and values what the other partners can contribute	
M4: Game changers—End-users and researchers find new and more productive ways of doing and implementing research through working in collaboration. They see wider potential for the new way of working	
M5: Facilitative leadership—Project teams are led by one or more leaders, who are regarded within and outside the team as credible and having real clout, connections, drive, enthusiasm, and tenacity. A facilitative style of leadership works well to involve partners, and to co-produce and mobilise knowledge for implementation	
M6: Small strategic core—Project teams are formed around a small strategic core of end-users and researchers from the partner organisations involved in the project	
M7: Creative assets—Partners harness existing and build up new assets to facilitate the conduct and implementation of AHR. ‘Assets’ include people with particular knowledge and skills; continuing professional development opportunities; routine data; websites for sharing learning; publications	
M8: Relational adaptive capacity—Learning from local AHR is actively shared with and adapted to kindred settings or populations in other areas (locally, nationally, internationally)	
M9: End-user is king!—Partners recognise that the key change agents are not the program ‘makers and shakers’ and the strategies they introduce but rather the agents on the ground and how they respond to the opportunities afforded by the program to change how AHR is routinely carried out and implemented	

Briefly, we found that whether the mechanisms were active or not in the individual projects reflected subtle but important differences in the ways in which the partners collaborated on the projects. Through these mechanisms, the partners on the more successful projects took advantage of the opportunities afforded by the programme within the different contexts of their work, some of which were more conducive and sensitive to their efforts than others. Based on these findings, we suggested that the style of closer collaboration that best enabled the partners to seize opportunities and overcome barriers to achieving knowledge translation could be construed as a form of ‘co-production’ of knowledge.

### Aims of the illustrative case study

In this paper, we develop and elaborate our thesis, addressing the question: what does the theory of co-production add to our understanding of the processes of knowledge creation and translation in PenCLAHRC? We begin by describing the theory and the core principles that underpin it. Using data from one of the projects where knowledge translation was readily achieved, we show how elements of co-production were encapsulated in the mechanisms of closer collaboration that were at play throughout the design, conduct and implementation of the project. We consider how the theory helps to explain why knowledge translation was achieved and how it might inform the future development and evaluation of collaborations in AHR more generally.

### The theory of co-production

Ostrom and colleagues propounded the idea of ‘co-production’ in the late 1970s [14]. She defined it as ‘a process through which inputs used to produce a good or service are contributed by individuals who are not “in” the same organisation’ (p. 1073) [14]. Subsequently, the concept has simply been used to describe people who ‘contribute to’ (p. 4) or ‘collaborate in’ (p. 16) the production of the public services that they use [14]. As we explain below, this means more than involving and engaging service users.

The notion of co-production is founded on a number of elements or principles. These have been variously defined in the literature, but we discerned five core elements. First, in the process of co-production, users are regarded as active agents and not merely passive subjects or recipients of services [14, 15]. Second, there is greater equality in the relations between users and professionals, with services becoming more user driven and users’ knowledge and experience being valued on a par with that of professionals [16–18]. Third, service users and professionals recognise that they can achieve more by working together than they can apart; both also find their relationship to be reciprocal and mutually beneficial [16, 18]. Fourth, users’ increased participation transforms the ways in which public services are designed and delivered, developing capacity for users’ present and emerging needs to be met [15, 16]. Fifth, the participation of users in the co-production of services is encouraged and facilitated by networks and organisations that support their involvement (although it is recognised that it is people, not systems, who create change) [15, 16].

As the above implies, the theory of co-production was originally developed to conceptualise a particular type of relations between the providers of goods or services (such as public officials) and users of them (citizens). However, in recent years, it has also been used to describe the growing engagement of policy makers and practitioners in applied research [19–21]. For example, Martin [20] outlined five types of practitioner engagement in research, which he described as ranging from relatively weak (‘practitioners as informants’) to strong examples (‘practitioners as co-researchers’) of co-production. This, in turn, prompted Nutley [21] to highlight issues for future debate and research on the topic, such as the appropriateness of the breadth of Martin’s typology, and whether the boundaries between the two communities of researchers and practitioners are maintained or collapsed in the course of the co-production of research.

To date, there has been only limited discussion of the relevance of the theory of co-production to the work of the CLAHRCs [13, 22]. In an interim report of their evaluation of the Birmingham and Black Country CLAHRC, Hewison et al. observed that although the programme was not formally conceived in terms of co-production at the outset, its approach to partnership working could be so characterised. They also noted that this way of working was taking longer than traditional approaches to AHR and that it remained to be seen if the desired outcomes would be achieved or not [22].

In contrast, the findings we report below are based on analysis that was carried out using data from the evaluation of the entire pilot of PenCLAHRC, when the initial outcomes of many of its projects were discernable. Through a case study of one of the projects where knowledge translation was readily achieved, we describe how the style of collaboration exemplified by the project can be interpreted as a form of co-production. We suggest that the theory of co-production provides a useful existing social theory for expounding the nature of the mechanisms that were characteristic of successful knowledge generation and translation projects in PenCLAHRC.

## Methods

The internal evaluation of PenCLAHRC involved semi-structured interviews with 54 programme stakeholders (some of whom were interviewed twice) and 28 members of four case study projects, as well as analysis of programme documents. Full details of the methods of data collection and analysis used are available elsewhere [13]. To elaborate the preliminary theoretical claims we made, we carried out further analysis of one of the research projects that exemplified the style of collaboration that was successful in bringing about knowledge translation. Our aim was to examine and illustrate in more depth the nature and extent of the correspondence between the mechanisms of closer collaboration and the principles of co-production, as manifest at different stages of the project.

### The stroke thrombolysis project case study

We selected the stroke thrombolysis project for more detailed analysis of our preliminary theoretical claims for three main reasons. First, the project was an early product of the system that PenCLAHRC had set up for soliciting research questions on topics that were important to the local community in the south-west of England. It was based on a question submitted by a stroke consultant in 2009 about the scope for, and potential benefits of, making changes to the existing emergency pathway for acute ischaemic stroke with the aim of minimising the time between the onset and treatment of the condition. The question was prioritised by PenCLAHRC and taken forward by a dedicated project team who worked up the idea, carried out the research in 2010–2011 (episodically) and implemented the findings locally by early 2012. The team included various clinicians and managers (from the stroke unit and emergency department in a local hospital and from the regional stroke network), paramedics (from the local regional ambulance trust), researchers with expertise in operational research methods and a project facilitator (all from the local university and PenCLAHRC).

Second, in our evaluation, we found the stroke thrombolysis project was a particularly successful example of what PenCLAHRC, and the national CLAHRC programme in general, was intended to achieve: the publication and implementation of evidence from high-quality research and increased capacity for AHR. Specifically, the research was published in *Stroke*, a leading journal [23]. It led to the existing emergency pathway for acute ischaemic stroke being redesigned and a pre-alert system being introduced by the ambulance and hospital trusts. Once implemented, there was a fourfold increase in the number of patients treated, in half the time previously taken. The project also helped to increase interest in and capacity for operational research in the local health economy. A number of similar projects were subsequently undertaken by a growing team of operational researchers in PenCLAHRC in partnership with NHS Trusts in the region.

Third, all nine of the mechanisms of closer collaboration that were associated, to varying degrees, with the more successful projects were found to be present and active in the case of the stroke thrombolysis project.

### Analysis

Further thematic analysis of qualitative data from the stroke thrombolysis project case study was carried out to enhance the original analysis. These data included semi-structured interviews, of 40–80-min duration, with nine participants involved in the project. They included five participants from the NHS Trusts and regional stroke network and four university and PenCLAHRC participants. The interviews were carried out between December 2012 and March 2013. Management briefings, conference papers and research publications from the project were also obtained and read for information about the conduct and impact of the work from its inception in 2009 to 2012. Over that period, the evaluation team was also engaged in wider observation of programme events and meetings that sometimes included a focus on the project.

We used a combination of concept mapping [24] and framework analysis [25] to examine if the mechanisms of closer collaboration discerned in the evaluation could potentially be explained in terms of the theory of co-production. Initially, we compared each of the mechanisms of closer collaboration (as specified in the evaluation) with the core elements of co-production (as defined in the existing literature on the topic) and identified potential links between these concepts. We then designed a matrix for cross-tabulating themes in the data relating to the five elements and their matching mechanisms (listed in rows), with the design, conduct and implementation phases of the stroke thrombolysis project (listed in columns). The resulting matrix captured evidence of the manifestation of the sorts of collaborative behaviour, actions or attitudes that were consistent (or not) with the principles of co-production at different stages in the research process.

JH led on this analysis. Entries in the matrix were independently checked by JD and NB against the data, who confirmed the initial entries and identified two additional examples of evidence supporting the links. The final versions of the matrix and conceptual map were agreed after review and discussion by all the authors.

## Ethics

We consulted the Chair of a NHS Local Research Ethics Committee who confirmed that, because the project was an evaluation, approval was not required. Participants were given information about the evaluation before the interviews and consented to the interviews being recorded and their views being reported anonymously.

## Results

**Fig. 1:**
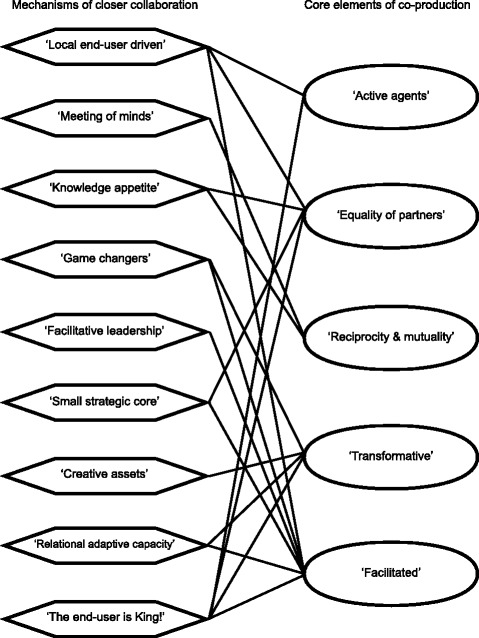
Concept map of the correspondence between mechanisms of closer collaboration and the core elements of co-production

In the concept mapping, each mechanism of closer collaboration was found to correspond with the qualities of one or more of the core elements of co-production (see Fig. 1). In the rest of this section, we examine the relevance of the theory, drawing on examples of how closer collaboration was performed throughout the stroke thrombolysis project. Quotations used to illustrate the analysis have been anonymised and slightly edited for presentation.

### Active agents

Central to ‘Engagement by Design’^©^ in PenCLAHRC was the idea that AHR should be primarily driven by the needs of end-users of the research, such as professionals in the NHS and service users, and not by researchers. This idea was explicit in the programme documents and in the stakeholders’ accounts of how PenCLAHRC was supposed to work. However, as our evaluation revealed, there was some variation in the extent to which the projects were perceived by members to be driven by end-users. Whether end-users drove the projects or not was found to be one of the nine mechanisms of closer collaboration that made a difference to the success of the projects (labelled ‘local end-user driven’ in Fig. 1). In the concept mapping, this and one other mechanism was found to be a good fit with the ways in which users were conceptualised to be ‘active agents’ in co-production, as we describe below.

The ‘local end-user driven’ mechanism was manifest throughout the design, conduct and implementation of the stroke thrombolysis project. For example, it was a stroke consultant who jointly conceived the idea for the project and who submitted it to PenCLAHRC for prioritisation. The same clinician also jointly led the project team throughout. The team included other users from the NHS who were involved in the emergency pathway for acute ischaemic stroke, namely senior clinicians from the hospital’s stroke unit and emergency department (ED), and paramedics from the local ambulance trust. Other clinicians, who were not part of the project team but who were involved in delivering the pathway, also collected bespoke information for the project and participated in a workshop organised by the researchers where they helped to build a computer simulation model of the existing pathway. These clinicians were also involved in piloting the pre-alert system that was introduced on the basis of modelling potential consequences of proposed service changes.

The other mechanism that fitted the concept of ‘active agents’ was one that the members of the stroke thrombolysis project, more than any of the projects examined in the overall evaluation, had consciously initialised in the course of their work. This was where the members were cognisant of the role of the clinicians on the ground, whom they recognised were ultimately the users who would accept and adopt (or not) any proposed change in the design of the pathway. The operation of this mechanism (‘the end-user is King!’) was apparent in the lead clinician’s assertion that it was important that the research was relevant and meaningful to these clinicians and their daily practice: ‘Well, I think the crucial thing is to always relate it [research] to real life patients and real life clinical practice…’ [ID1]. The researchers accordingly carried out additional modelling in an attempt to engage and assuage these professionals and to demonstrate the ‘real-life’ problems that the research was addressing.

Although the team worked hard to involve various clinicians in the process of building the computer model of the pathway, the researchers realised during the project that they had not involved a wide enough range of clinicians from the ED early enough in the conduct of the research. This came to light during a meeting with the ED staff where the project team encountered some concerns about, and resistance to, some of the assumptions underpinning the model that they had constructed to date. The team were able to address these concerns but recognised that the scenario could have been avoided if they had involved a wider range of professionals earlier in the process. This experience only confirmed their view that it was important to include representatives of all the relevant professionals in the process of building a model, to make it sufficiently realistic and trustworthy, and to increase the chances of the results being accepted by them and acted upon.

### Equality of partners

By seeking to enable clinicians, patients and the public to play a more active role in AHR, PenCLAHRC was also encouraging them to have a bigger and more equal role in the research process. Thus, the two aforementioned mechanisms (‘local end-user driven’ and ‘the end-user is King!’) were also found to fit with the ‘equality of partners’ principle of co-production.

Another mechanism that made a difference to the success of the projects in general was the size and composition of the project teams (‘small strategic core’). In the case study project, a stroke consultant (who was also an active clinical academic) and a senior researcher jointly led the project. They had worked together before and trusted and valued each other. The team also included paramedics from a local ambulance trust who had not collaborated before with the other members prior to the establishment of PenCLAHRC. For one of them, an unexpected benefit of the project was the development of working relationships that went beyond it:

‘I think success is often dependent on good working relationships and networking and we’ve certainly done that you know I’ve almost got a friend in [operational researcher X] now and I know I can email [X] with any queries now whether it’s about this pathway or any other thing you know “I’ve got this idea and can I run it by you” and he is more than happy to help and support and [Y] is a fantastic lead … now I am on personal terms with [Y] before I may have been a little hesitant about emailing [them] but I think we’ve got quite a good relationship now.’ [ID5]

The rest of the project team was small but stable throughout and inclusive of the key clinicians and researchers from the relevant partner organisations, each of whom were well positioned to progress the research. All the members were clear about their roles, which were distinct and vital to the success of the project. In these respects, we found the inclusiveness and even distribution of power in the project fitted with the general ‘equality of partners’ principle of co-production.

Whether the various clinicians in the PenCLAHRC projects valued the researchers’ different knowledge and expertise (and vice versa), was also found to be a key mechanism (‘knowledge appetite’). In the stroke thrombolysis project, the lead clinician was initially unfamiliar with the operational research methods that the researchers proposed to use but quickly saw the relevance of the approach:

‘I had little understanding before I started on this about what operational research was or what it could do … the crucial thing about the collaboration, as soon as I was put in touch with people who knew how to do this, everything fell into place from my point of view very quickly, because I had a clear idea of what a clinician would want from that sort of project, and [operational researchers] had a very clear idea of what operational research had to offer that sort of work. So to me it clicked very quickly.’ [ID1]

Likewise, the researchers were interested in finding out from the clinicians how the local emergency pathway for acute ischaemic stroke worked in practice, in order to be able to model it and estimate the effects of changing it. The clinicians who helped to build the model all reported that they felt that their contribution was valued by the researchers, and they very much enjoyed the process of taking part. In these respects, the knowledge and expertise of all the members were equally valued in the team.

### Reciprocity and mutuality

Whether the different partners on the projects were open to and interested in learning from each other (‘knowledge appetite’) was also found to link to the ‘reciprocity and mutuality’ element of co-production. This was because the partners recognised that they needed each other’s knowledge and experience to meet the aims of the project and found that they each benefitted from being involved in the collaboration.

In the stroke thrombolysis project, this was evident in the researchers’ need for good-quality routine data for the modelling to be feasible. After being denied access to one potential source of data, the researchers were able to access another with the help of the lead clinician:

‘I think we never would have got it [data] as academics we never would have got hold of it, it was only through [clinician’s] influence that we were able to get access to the data. So [clinician] was fundamental’ [ID3]

Through combining their knowledge and connections, the partners found that they achieved both their primary shared aim and other distinctive organisational goals. For example, the NHS Trusts and patients benefitted from implementation of the findings, which improved the emergency pathway for acute ischaemic stroke and reduced disability. The university benefitted from publishing the work in international journals and by achieving impact that was directly attributable to the research.

In addition, whereas in some of the projects in PenCLAHRC, the aims shifted over time or were never settled and agreed at all levels in their respective organisations, in the stroke thrombolysis project, the various clinicians and researchers all agreed about the aims of the research and the methods to be used (‘meeting of minds’). The project team members also had the full support of their respective organisations at a senior level and on the ground (after the concerns of some of the ED staff were addressed).

In reflecting on what they got out of the project, one of the stroke clinicians also observed that their involvement in this type of project provided a way of learning about each other’s part in the pathway:

‘It’s just been a really good positive project, it’s been a real interesting way to see process mapping applied to a clinical process incorporating very complex processes and organisations because we all are and we all work independently but looking at how we can pull them together with one common motorway if you like and it’s been very good I’ve enjoyed it’ [ID8]

This also led to an improvement in the working relationships between the stroke and ED departments, with the stroke clinicians feeling that their role was valued more as a result of what the model showed.

### Transformative

As noted, a fundamental aim of the CLAHRCs was to transform the ways in which AHR was conducted. In PenCLAHRC, the researchers and clinicians in some of the projects found that their experience of working in collaboration on the projects was different to how they had carried out research before (‘game changers’) and opened up new possibilities and capacity. The operation of this mechanism was particularly evident in the case study project and found to fit with the ‘transformative’ principle of co-production. For example, the lead clinician reflected that

‘And what I find myself doing now, having had experience of the collaboration and the operational research, is when I look at other clinical problems that colleagues describe to me I end up looking at that and thinking, well, actually what you need is not do what the NHS has done before, which is muddle through on the strength of inadequate data through a process of trial and error … what you need to do is organise an operational research project … And, in fact, that’s the way we’ve done it with some of the spin-out projects’ [ID1]

The clinician also reported finding it easier to ‘sell’ the project to clinical colleagues, and to make a case for the proposed redesign of the emergency pathway for acute ischaemic stroke, using the evidence from the researchers’ models rather than having to rely on ‘hunches’ or suppositions based on experience, as before. And as we mentioned earlier, the researchers claimed that without the help of the lead clinician, they would not have been able to obtain access to the data that they needed for the modelling. By working directly and immediately with the clinicians and their organisations to help them model the pathway and implement service changes, the researchers were also able to see the outcomes of the work, which they personally found more satisfying than not being involved in the implementation phase of the research.

As noted above, the researchers’ recognition that they needed to engage a full range of clinicians in future projects in order to enhance the credibility and acceptability of the modelling to those who would be involved in implementing any proposed changes (‘the end-user is King!’) also fitted with the ‘transformative’ element of co-production.

So, too, did the ways in which members of the team pooled and utilised each other’s local and specialised knowledge, resources and connections (‘creative assets’). For example, members were able to draw on existing data, specialist research methods, connections with colleagues and networks and the clinicians’ mundane knowledge of the day-to-day workings of the emergency pathway for acute ischaemic stroke, to effectively deliver the project. Paramedics from the ambulance trust subsequently became involved with other PenCLAHRC operational research projects after their positive experience of collaborating on the stroke thrombolysis project.

Members of the team were also subsequently enabled by PenCLAHRC to visit other centres in the region and demonstrate the potential of applying the approach to local variations of the emergency pathway for acute ischaemic stroke and configurations of services in these trusts. Through this mechanism (‘relational adaptive capacity’), the members endeavoured to promote the methodology and findings of the research to clinicians in other settings in the south-west of England.

### Facilitated

Finally, several of the mechanisms of closer collaboration already mentioned above were also found to fit with the ‘facilitated’ principle of co-production. This was where PenCLAHRC and/or the individual projects had structures or procedures that supported this style of collaboration. The first of these concerned the ways in which some of the PenCLAHRC projects were led (‘facilitative leadership’). In the stroke thrombolysis project, the joint clinical and research leads were both perceived by the rest of the team to have the relevant qualities of being credible, enthusiastic and inclusive in their approach. They were also regarded as having good contacts within and outside their organisations and being well placed to progress the research. For example, the lead clinician had strong links with the local and national stroke research networks and organisations, and the researchers had established connections with colleagues in other universities who advised on some aspects of the modelling.

The size and composition of the project teams (‘small strategic core’) was also perceived by members of the stroke thrombolysis project team to have facilitated the research. The inclusive and participatory nature of the methods used by the researchers to build the computer model, and the extra modelling they carried out for some clinicians, also helped to engage the relevant clinicians in the process of the research through to the implementation stage (‘the end-user is King!’). As one of the operational researchers observed, this was a recognised aspect of how they worked:

‘..the general ethos of operational research is it’s important to involve stakeholders within the process of building a model if you’re going to sort of improve the chances of implementation’ [ID4]

More generally, PenCLAHRC provided an infrastructure that enabled and supported the stroke thrombolysis and other projects. For example, it established the process by which the stroke consultant was able to submit a question for prioritisation; it also funded some of the lead clinician’s time for working on the project, enabling them to be involved in all stages of the research process (‘local end-user driven’). By bringing together clinicians, researchers, and project facilitators and support staff, PenCLAHRC also provided its partners with an opportunity to do AHR in a different way and to systemically generate new projects between partners (‘game changers’). Finally, as described above, PenCLAHRC also provided funding to enable the members of the stroke team to visit other centres in the south-west to promote the methodology and encourage clinicians in other centres to follow suit (‘relational adaptive capacity’).

## Discussion

This analysis was undertaken to examine what the theory of co-production might add to our understanding of the processes by which knowledge is produced and utilised in health care through research collaborations. Like Hewison and colleagues [22], we found that the concept of co-production was not explicit in PenCLAHRC’s programme discourse. Despite this, there was a strong correspondence between the principles that underpin the theory and the various mechanisms of closer collaboration that were in operation in the more successful projects in PenCLAHRC.

In the stroke thrombolysis project, the co-production ethos was manifest throughout the research process, from the conception of the project through to the local implementation of the findings—and potentially beyond through the team’s efforts to further translate the modelling of the emergency pathways for acute ischaemic stroke elsewhere in the south-west. This continuity further distinguishes co-production from other episodic forms of collaboration that are confined to particular stages of the research process, such as the commissioning or dissemination phases, or occasional points where advisory groups are engaged in the process.

In contrast to Martin’s typology [20], where the most active form of co-production involved clinicians acting as ‘co-researchers’ (p. 217), we found that the clinicians were actively involved in the project in their capacity as ‘clinicians’ and ‘clinical academics’. That is, they contributed their own distinct expertise, experience and assets to the collaboration. Although there was some blurring of roles, the clinicians and researchers still retained their respective identities and distinct professional positions and objectives. This distinction is consistent with what Van de Ven and Johnson [26] refer to as ‘engaged scholarship’ (p. 803) where scholars and practitioners from diverse backgrounds pool their respective knowledge and expertise to exercise more leverage over social problems.

While we found some projects progressed slowly in PenCLAHRC, this was for various different reasons (and not just the time it took to develop partnerships in individual projects). In the stroke thrombolysis project, there was evidence that the engagement of clinicians in the project had enabled the team to overcome potential difficulties and avoid delays, such as helping to source and access alternative datasets, without which the project might have stalled or failed completely. However, the greater involvement of clinicians also introduced some additional complexity for the researchers, leading to some co-authored publications taking a little longer to be produced than might otherwise have been the case. This highlights a key point that the variable progress and success of the projects in PenCLAHRC depended on the complex interplay of the various mechanisms of closer collaboration in the contexts of the individual projects and the wider programme.

Through a combination of the mechanisms described in the paper, the members of the stroke thrombolysis project were able to take advantage of the opportunities afforded by the programme and overcome the inertia of the system and local barriers to introducing a change in the emergency pathway for acute ischaemic stroke. However, the efforts of the team may well have been thwarted and insufficient if, say, the style of leadership was different, or a suitable alternative dataset did not exist, or the methodology was less participatory, or the clinicians from the ED had undermined the model, or the results of the modelling had shown that a clinical role in the emergency pathway was superfluous (thereby possibly losing the said clinicians’ engagement with the work). In other words, whether a given collaborative research project was successful or not depended on how it was enacted by the partners involved in the local and wider contexts in which it was situated and how conducive and sensitive those contexts were to the efforts of the team.

An important implication of this finding for those involved in designing future CLAHRC-like programmes is that they could do more to promote not just collaboration per se, but a particular style of collaboration, such as one founded on the principles of co-production, the elements of which we have described in this paper. When the CLAHRCs were originally set up in England, as nine local experiments in knowledge translation, they were encouraged to try different approaches and no particular approach was privileged. While we are not suggesting that the co-productive approach is a panacea, it did help some participants in PenCLAHRC projects to overcome barriers in their local contexts of operation and meet their goals. Programme architects could help to foster this particular approach by explicitly embedding the principles of co-production into the raison d’être and operating frameworks of future programmes of applied health research.

While the PenCLAHRC infrastructure clearly helped to facilitate closer collaboration between researchers and clinicians in the south-west of England, and to achieve clinical goals in some projects, the introduction of the programme was not in itself sufficient to make this happen. Successful AHR ultimately depended on how programme and project members and subjects interacted and utilised ideas and resources available to them, while working in contexts where different factors variously supported and impeded their efforts. The value of the theory of co-production is that it recognises this in its core principles and, in particular, in the idea that end-users’ participation is critical to the successful creation and utilisation of services and goods, including knowledge.

### Limitations

We deliberately focussed here on a case of successful knowledge translation identified from a wider evaluation [13] in order to examine and illustrate in more depth the style of collaboration it exemplified. Although the featured stroke thrombolysis project itself involved a small number of interviews, the analysis builds on related findings from a thematic examination of interviews with 28 members of four projects and documentary analysis that were carried out as part of the wider evaluation.

A limitation of this work is that it only examined the collaborative relations between clinicians and researchers in PenCLAHRC and not the contribution of patients and the public, who played a significant part in around half of the projects undertaken during the pilot programme. In the interviews with members of the four project teams, we did ask about the actual or potential contribution of patients and the public. The stroke thrombolysis team found it hard to see what patient or public members might have contributed to this particular project, but the researchers were open to the possibility of involving them in future operational research projects and have done so in subsequent work in PenCLAHRC [27].

## Conclusions

We found a close fit between the nine mechanisms of closer collaboration at work in the project and the principles of co-production. The successful style of collaborative working exemplified by the project was consistent with a strong form of co-production. In our view, the theory of co-production provides useful insights into what it is about the qualities of collaborative working that inspire the requisite mechanisms for generating knowledge that is translated into practice.

Future research is required to examine the nature, challenges, benefits, and pitfalls of collaborative research developed in accordance with the principles of co-production in different contexts and timescales. In particular, there is a need to examine these issues in collaborations in AHR where the third community of patients and the public are also engaged alongside clinicians and academics throughout the research process. There is also a need to examine whether and how health care research that is co-produced in one locality can be effectively translated to other settings through some process of connected co-production involving extended groups of end-users.
